# Bibliographical

**Published:** 1890-12

**Authors:** 


### Bibliographical.

We have just received a compend of dental pathology and dental medicine containing most noteworthy points on the subjects of interest to the dental students, by Geo. W. Warren, D.D.S., Clinical Chief Pennsylvania College of Dental Surgery. Illustrated.

This is a quiz compend just issued from the press of P. Blakeston, Son & Co., of Philadelphia. It is a most excellent little work for the use of the student in regard to the subjects upon which it touches, namely: anatomy and physiology dental medicine, weights, measures, etc. It is very valuable to the student, being very compact and clear in its statement upon the various subjects presented. The examination of such a work as this brings strongly to mind the thought that much of our literature is overloaded with verbiage. Every writer should make an effort as much as in him lies to condense the most thought into the fewest words and smallest space.

This little work is remarkable for its absence of technical terms in which some seem to take so much delight. This little work should be in the hands of every dental student, and every practitioner, as well, would be the better for having and consulting it.

THE percentage of graduates among the physicians in Illinois was only 48 per cent. before the registration law went into effect; now it is 91 per cent.

WE are gratified to learn that at the recent commencement of the Fort Wayne College the degree of A.M. was conferred upon Dr. S. B. Hartman, of that city.

The doctor has been a persevering student and engaged in much research and investigation in science and literature. The honor conferred upon him was upon real merit, and because it was deserved. It was done without his previous knowledge and of course is highly appreciated. He is also a graduate of the Dental Department of the University of Michigan, being a member of its senior class in the second year of that department. It is always gratifying to learn of this kind of progress and attainment by members of our profession. It is practicable for almost every man to make himself complete master of some one or more subjects, and such attainment always gives him an advance position over others who are content to remain at a common level in all things.

